# Supplementary material for: Analysis of PPARγ Signaling Activity in Psoriasis
Source: Int J Mol Sci. 2021 Aug 10;22(16):8603. doi: 10.3390/ijms22168603 (PMC8395241; doi:10.3390/ijms22168603)
Supplement: Supplementary file 1 [file ijms-22-08603-s001.zip › Supplemental materials_Analysis of PPARg signaling activity in psoriasis/Pathway models/Models images and html files/Differentiation of psoriatic T cells/6311.html]

IL1B


# Protein IL1B

|  |  |
| --- | --- |
| URN | urn:agi-llid:3553 |
| Total Entities | 0 |
| Connectivity | 18723 |
| Name | IL1B |
| Description | interleukin 1, beta |
| Notes | The protein encoded by this gene is a member of the interleukin 1 cytokine family. This cytokine is produced by activated macrophages as a proprotein, which is proteolytically processed to its active form by caspase 1 (CASP1/ICE). This cytokine is an important mediator of the inflammatory response, and is involved in a variety of cellular activities, including cell proliferation, differentiation, and apoptosis. The induction of cyclooxygenase-2 (PTGS2/COX2) by this cytokine in the central nervous system (CNS) is found to contribute to inflammatory pain hypersensitivity. This gene and eight other interleukin 1 family genes form a cytokine gene cluster on chromosome 2. [provided by RefSeq, Jul 2008] |
| Primary Cell Localization | Extracellular |
| Class | Ligand |
| ObjectType | Protein |

---

|  |  |
| --- | --- |
| Pathway | Alzheimer's Disease |
|  | Osteoarthritis |
|  | Toll-like Receptors Act through MYD88-TIRAP Signaling |
|  | NOD-like Receptors |
|  | Dectin-1 (CLEC7A) Signaling |
|  | TLR4 Signaling in Leukocytes |
|  | Th17-Cell Differentiation |
|  | Plasmin Effects in Inflammation |
|  | Bradykinin Effects in Inflammation |
|  | Toll-like Receptors in Sterile Inflammation |
|  | Toll-like Receptor Independent Sterile Inflammation |
|  | Hypothalamic-Pituitary-Adrenal Axis in Acute Phase Response |
|  | MC1R in anti-Inflammatory Signaling |
|  | Lipids Enhance Apoptotic Cell Engulfment and Reduce Inflammation |
|  | Keep out Signal: LTF Inhibits Neutrophil Recruitment and Inflammation |
|  | Prostaglandin E2 Receptor Signaling in Neurons |
|  | Macrophage Related Iron Uptake and Release |
|  | Macrophage M1 Lineage |
|  | anti-Inflammatory Function of Macrophage M2 Lineage |
|  | Negative Acute Phase Proteins Synthesis |
|  | Positive Acute Phase Proteins Synthesis |
|  | Vascular Endothelial Cell Activation by Cytokines |
|  | Vascular Endothelial Cell Activation by Blood Coagulation Factors |
|  | Adipokines Production by Adipocyte |
|  | Nociception Expression Targets Signaling |
|  | CNR1/2 -> IL1B/2/4/6/10 Production |
|  | IL1B Expression Targets -> Nociception |
|  | Proteins Involved in Atherosclerosis |
|  | Proteins Involved in Arterial Hypertension |
|  | Proteins Involved in Pulmonary Hypertension |
|  | Lymphocyte Mediated Myocardial Injury in Myocarditis |
|  | Proteins Involved in Myocarditis |
|  | Toll-like Receptors (TLR) in Antiviral Innate Immune Response |
|  | Inflammatory Reaction in Acne Vulgaris |
|  | Proteins Involved in Atopic Dermatitis |
|  | Proteins Involved in Psoriasis |
|  | T-Cells Differentiation Block in Psoriasis |
|  | Dendritic Cells Function in Psoriasis |
|  | Apoptotic Keratinocytes Clearance Recession in Systemic Lupus Erythematosus |
|  | Dendritic Cell Activation in Systemic Lupus Erythematosis |
|  | Th17-Cell Function in Systemic Lupus Erythematosus |
|  | Vitiligo |
|  | T-Cell Cytotoxic Response against Melanocytes in Vitiligo |
|  | Plasmacytoid Dendritic Cell in Diabetes Mellitus Type 1 |
|  | Toll-like Receptors in beta-Cell |
|  | Macrophage Function in Diabetes Mellitus Type 1 |
|  | Proteins Involved in Diabetes Mellitus Type 1 |
|  | Conventional Dendritic Cell Role in Diabetes Mellitus Type 1 |
|  | Proteins Involved in Diabetes Mellitus Type 2 |
|  | Proteins Involved in Diabetic Neuropathy |
|  | Proteins Involved in Insulin Resistance |
|  | Proteins Involved in non-Alcoholic Fatty Liver Disease |
|  | beta-Cell Destruction through Cytokines in Diabetes Mellitus |
|  | beta-Cell Destruction in Diabetes Mellitus Type 1 |
|  | Nitric Oxide Effects on beta-Cell |
|  | Apoptosis of Thyroid Cell in Hashimoto's Thyroiditis |
|  | Proteins Involved in Graves Disease |
|  | Graves Ophthalmopathy |
|  | Proteins Involved in Hypothyroidism |
|  | Fatty Acids in Hypertension and Atherosclerosis Development |
|  | Adipokines Production by Adipocyte Impaired in Obesity |
|  | Proteins Involved in Obesity |
|  | CD4+T-Cell Response in Celiac Disease |
|  | Crohn's Disease |
|  | Paneth-Cell Function in Crohn's Disease |
|  | Macrophage Dysfunction |
|  | Proteins Involved in Inflammatory Bowel Diseases |
|  | Dendritic Cell Dysfunction in Crohn's disease |
|  | Proteins Involved in Helicobacter Infections |
|  | Dendritic Cell Function in Ulcerative Colitis |
|  | Leukocyte Migration toward the Endothelial Cell in Ulcerative Colitis |
|  | Nicotine anti-Inflammatory Effect in Ulcerative Colitis |
|  | Proteins Involved in Ulcerative Colitis |
|  | Proteins Involved in Multiple Myeloma |
|  | Proteins Involved in Thrombophilia |
|  | Proteins Involved in Pancreatic Cancer |
|  | TRPM7/8 in Pancreatic Adenocarcinoma |
|  | TSLP Signaling in Bronchial Epithelial Cell in Asthma |
|  | Goblet-Cell Related Mucus Secretion in Asthma |
|  | Antigen-Presenting Cell Role in Asthma |
|  | Th17-Cell Differentiation in Asthma |
|  | CFTR Facilitates Oxidative Stress in Airway Epithelium in CF |
|  | Proteins Involved in Cystic Fibrosis |
|  | Airway Epithelial Cell Dysfunction |
|  | CFTR Expression in Epithelial Cells (Class I Mutations) |
|  | Proteins Involved in Gout |
|  | Proteins with Altered Expression in Gout |
|  | Osteoclast Activation in Gout |
|  | Neutrophil Recruitment in Sinovium in Gout |
|  | Monocytes Function in Gout |
|  | TNF and IL1B Induce Metalloproteinase Synthesis in Osteoarthritis |
|  | IL1B Induced Arthralgia |
|  | Proteins Involved in Osteoarthritis |
|  | Dendritic Cell Dysfunction in Psoriatic Arthritis |
|  | Th17-Cell and Th1 Immune Responsein Psoriatic Arthritis |
|  | Synovial Fibroblast Activation in Psoriatic Arthritis |
|  | Proteins Involved in Psoriatic Arthritis |
|  | Synovial Fibroblast Proliferation in Rheumatoid Arthritis |
|  | Osteoclast Activation in Rheumatoid Arthritis |
|  | Synovial Fibroblast Activation by Citokines in Rheumatoid Arthritis |
|  | TLR2 Induced Synovial Fibroblast Activation in Rheumatoid Arthritis |
|  | Proteins Involved in Rheumatoid Arthritis |
|  | Autocrine Cytokine/Chemokine Loops in Systemic Scleroderma |
|  | FAS Mediated Apoptosis in Systemic Scleroderma |
|  | Proteins Involved in Systemic Scleroderma |
|  | Proteins Involved in Dental Caries |
|  | Proteins Involved in HIV Type 1 Infection |
|  | Th17-Cell Numbers Reduction in HIV |
|  | CD4+ T-Cell Function Decline in HIV |
|  | Toll-like Receptors (TLR) in Periodontitis |
|  | Diabetes Induced Periodontitis |
|  | Proteins Involved in Periodontitis |
|  | IL1 Receptor Antagonist (IL1RN) Deficiency (DIRA) |
|  | PAPA Syndrome |
|  | TNF Receptor Associated Periodic Syndrome |
|  | Mevalonate Kinase Deficiency |
|  | Blau Syndrome |
|  | IL36 Receptor Antagonist (IL1F5) Deficiency (DITRA) |
|  | Cryopyrin (NLPR3) Associated Periodic Syndromes |
|  | Familial Cold Autoinflammatory Syndrome 2 |
|  | Familial Mediterranean Fever |
|  | Majeed Syndrome |
|  | NF-kB Signaling Activation in Huntington Disease |
|  | Muscular Dystrophies |
|  | Proteins with Altered Expression in Osteoarthritis |
|  | Proteins Involved in Osteoporosis |
|  | Proteins with Altered Expression in Rheumatoid Arthritis |
|  | Microglia Activation in Alzheimer's Disease |
|  | Proteins Involved in Alzheimer's Disease |
|  | Proteins Involved in Epilepsy |
|  | Ketogenic Diet Mechanism (Hypothesis) |
|  | Blood-Brain Barrier Disruption in Epileptiform Disorders |
|  | HMGB1 and IL1B in Neuroinflammation |
|  | ADK Expression Downregulation after Acute Seizures |
|  | HMGB1 and IL1B in Neuronal Hyperexcitation |
|  | Proteins Involved in Glioblastoma |
|  | Proteins Involved in Astrocytoma |
|  | Proteins Involved in Glioma |
|  | Proteins Involved in Neuroblastoma |
|  | Microglia Activation in Parkinson's Disease |
|  | Proteins with Altered Expression in Parkinson's Disease |
|  | Proteins Involved in Parkinson's Disease |
|  | Proteins with Altered Expression in Amyotrophic Lateral Sclerosis |
|  | Proteins Involved in Amyotrophic Lateral Sclerosis |
|  | Microglia and Motor Neuron Interaction Dysregulation |
|  | Neuroinflammation in Amyotrophic Lateral Sclerosis |
|  | Proteins Involved in Multiple Sclerosis |
|  | Proteins Involved in Sleep Initiation and Maintenance Disorders |
|  | Proteins Involved in Sleep Disorders |
|  | Proteins Involved in Adhesion, Migration and Invasion of Endometriotic Cell |
|  | Local Estrogen Production in Endometriosis |
|  | Proteins Involved in Endometriosis |
|  | Proteins with Altered Expression in Endometriosis |
|  | Leptin Role in Endometriosis |
|  | Proteins Involved in Spontaneous Abortion |
|  | Subepithelial Fibroblasts in Middle Ear Cholesteatoma |
|  | HPV E6 and E7 Induced Disruption of TNFR/NF-kB Signaling in Keratinocytes |
|  | Androgen Receptor/NKX3-1 Signaling |
|  | Proteins Involved in Age-Related Macular Degeneration |
|  | Proteins Involved in Hearing Loss |
|  | Cochlear Inflammation (Noise-Induced) |
|  | Proteins Involved in Otitis Media |
|  | Cytokines Trigger Otitis Media |
|  | Immune System Role in Otitis Media |
|  | Alveolar Macrophage Negative Regulation Declined in COPD |
|  | Proteins Involved in Bronchiectasis |
|  | Alveolar Macrophages Dysfunction in COPD |
|  | Proteins Involved in Chronic Obstructive Pulmonary Disease |
|  | T-Cell Cytotoxic Activation |
|  | Alveolar Epithelial Cell Dysfunction in COPD |
|  | Mucin Hyperproduction in Goblet and Mucous Cells |
|  | Proteins Involved in Glomerulonephritis |
|  | Proteins with Altered Expression in Glomerulonephritis |
|  | Plasmin Role in Glomerulonephritis |
|  | Endothelial Cell Dysfunction in Glomerulonephritis |
|  | Proteins with Altered Expression in Pyelonephritis |
|  | Toll-like Receptors in Pyelonephritis |
|  | Endothelial Cell Dysfunction in Pyelonephritis |
|  | HMGB1 Expression Targets |
|  | S100B Expression Targets |
|  | AREG -> HIF1A Expression Targets |
|  | AREG -> AP-1 Expression Targets |
|  | Noradrenaline/Gs Expression Targets |
|  | Dopamine/Gs Expression Targets |
|  | Noreadrenaline/Gq Expression Targets |
|  | Epinephrine/Gs Expression Targets |
|  | Dopamine/Gi Expression Targets |
|  | BMP7/BMPR2/ACVR2 Expression Targets |
|  | Elevated Receptors -> Expression Targets in Adipose Tissue |
|  | Elevated Receptors -> Expression Targets in Joint |
|  | CD8 -> ATF/CREB/CREBBP Expression Targets |
|  | CD8 -> STAT Expression Targets |
|  | CXCL3 Expression Targets |
|  | CXCL12 Expression Targets |
|  | CSF2 -> STAT Expression Targets |
|  | EGF -> CTNN Expression Targets |
|  | EGF -> AP-1/ATF Expression Targets |
|  | EGF -> CREB/CREBBP/ELK/SRF/MYC Expression Targets |
|  | EGF -> MEF/MYOD/NFATC Expression Targets |
|  | EGF -> STAT Expression Targets |
|  | F2 -> AP-1/CREB/ELK/SRF/SP1 Expression Targets |
|  | F2 -> STAT1/NF-kB Expression Targets |
|  | PLG -> AP-1/CREB/ELK/SRF/SP1 Expression Targets |
|  | PLG -> STAT1/NF-kB Expression Targets |
|  | FGF18 -> STAT Expression Targets |
|  | FGF18 -> AP-1/CREB Expression Targets |
|  | VIP Expression Targets |
|  | Elevated Receptors -> Expression Targets in Small Intestine |
|  | Elevated Receptors -> Expression Targets in Mouth |
|  | GH1/GHR -> NF-kB/ELK/SRF/MYC Expression Targets |
|  | GH1/GHR -> STAT Expression Targets |
|  | GH1/PRLR Expression Targets |
|  | TAM Receptor Signaling Activation in Cancer |
|  | STAT3 and NFkB Activate Inflammation-Induced Tumorigenesis |
|  | IL23A and IL17A Provoke Cancer-Associated Inflammation |
|  | Asbestosis- and Silicosis-Associated Inflammation Provokes Cancer Development |
|  | Proteins with Altered Expression in Tumor-Promoting Inflammation |
|  | N2 Neutrophils in Tumor-Promoting Inflammation and Tumor Progression |
|  | VEGFA Dependent Angiogenesis in Cancer |
|  | Tumor Infiltrating Macrophages in Cancer Progression and Immune Escape |
|  | IDO1 in Cancer Immune Escape |
|  | Fibronectin Expression Targets |
|  | Fibrinogen Expression Targets |
|  | NOTCH -> Expression Targets in Skin |
|  | IFNG/IFNR Expression Targets |
|  | IL1R -> STAT3 Signaling |
|  | IL1R -> NF-kB Signaling |
|  | IL13 Expression Targets |
|  | IL16 -> AP-1 Expression Targets |
|  | IL3 Expression Targets |
|  | IL4 Expression Targets |
|  | IL2 Expression Targets |
|  | IL16 -> NF-kB Expression Targets |
|  | Leptin -> STAT Expression Targets |
|  | Leptin -> ELK/SRF Expression Targets |
|  | LPA Expression Targets |
|  | PAF/Gq -> AP-1/ATF1/CREB/ERK/SRF Expression Targets |
|  | S1P Expression Targets |
|  | Proteins/Ions Transporters -> Expression Targets in Lymphoid System and Blood |
|  | Elevated Receptors -> Expression Targets in Lymphoid System and Blood |
|  | F2R/F2RL1 -> Expression Targets in Lymphoid System and Blood |
|  | GPCRs Family -> Expression Targets in Lymphoid System and Blood |
|  | Cell Adhesion Receptors -> Expression Targets in Lymphoid System and Blood |
|  | TNF Receptors -> Expression Targets in Lymphoid System and Blood |
|  | Immunoglobulin Superfamily -> Expression Targets in Lymphoid System and Blood |
|  | Elevated Receptors -> Expression Targets in Bone Marrow |
|  | C5AR1 -> Expression Targets in Lymphoid System and Blood |
|  | IgE Receptors -> Targets in Lymphoid System and Blood |
|  | Elevated Receptors -> Expression Targets in Thymus |
|  | CNR1-> Expression Targets in Nerve Tissue |
|  | GPCRs -> Expression Targets in Brain |
|  | P2RX7 -> Expression Targets in Nerve Tissue |
|  | Elevated Receptors -> Expression Targets in Nerve Tissue |
|  | BDNF Expression Targets |
|  | OSM/OSMR Expression Targets |
|  | PDGF -> AP-1/CREB/CREBBP/MYC Expression Targets |
|  | PDGF -> STAT Expression Targets |
|  | NTS Expression Targets |
|  | POMC Expression Targets |
|  | IFNA1/Gq Expression Targets |
|  | TAC1 Expression Targets |
|  | PRL/PRLR Expression Targets |
|  | PRL/GHR -> STAT Expression Targets |
|  | PRL/GHR -> NF/kB/ELK/SRF/MYC Expression Targets |
|  | CSF1 -> STAT Expression Targets |
|  | KITLG -> STAT Expression Targets |
|  | KITLG -> AP-1/CREB/CREBBP/MYC Expression Targets |
|  | PAF Expression Targets |
|  | CSF1 -> AP-1/CREB/CREBBP/MYC Expression Targets |
|  | PGF -> AP-1/CREB/CREBBP/MYC Expression Targets |
|  | MSTN/ACVR2/BMPR Expression Targets |
|  | Elevated Receptors -> Expression Targets in Lung |
|  | TCR -> AP-1 Expression Targets |
|  | TGFA -> STAT Expression Targets |
|  | TGFA -> CTNNB/CTNND Expression Targets |
|  | TGFA -> HIF1A Expression Targets |
|  | TGFA/AP-1/ATF Expression Targets |
|  | TGFA -> CREB/CREBBP/ELK-SRF/MYC Expression Targets |
|  | TGFB1-TGFBR2 Expression Targets |
|  | TGFB1-TGFBR1/AP-1 Expression Targets |
|  | TGFB1-ACVRL1 Expression Targets |
|  | FASLG Expression Targets |
|  | TNF -> NF-kB Expression Targets |
|  | TNF -> AP-1 Expression Targets |
|  | CD72 -> AP-1 Expression Targets |
|  | CD40LG -> NF-kB/ELK/SRF -> CREB/NFATC Expression Targets |
|  | IL1B -> NO Expression Targets |
|  | IL1B -> PGE2 Expression Targets |
|  | TLR1 -> 2/6 Expression Targets |
|  | IL1B Expression Targets |
|  | TLR4 -> NF-kB/IRF Expression Targets |
|  | IL1A Expression Targets |
|  | TLR9 Expression Targets |
|  | Dioxin Induced Chloracne (Hypothesis) |
|  | WNT5A Expression Targets |
|  | e |
|  | pathway 4 |
|  | New Pathway (2) |
|  | PPAR Psoriasis |
|  | pprarg neg, uknown targets, ps-positive |
|  | prarg neg,ukn expres targets, ps-positive |
|  | PPARG negative targets |
|  | pparg expr target\_regulators |
|  | Neighbors of disease exacerbation |
|  | Neighbors of tumor progression |
|  | Neighbors of cancer progression |
|  | Neighbors of inflammatory status |
|  | Neighbors of overall survival |
|  | Neighbors of disease severity |
|  | Neighbors of tumor size |
|  | Neighbors of lesion size |
|  | Neighbors of mortality |
|  | Neighbors of survival rate |
|  | Neighbors of infarct size |
|  | Neighbors of survival time |
|  | Neighbors of functional recovery |
|  | Neighbors of insulin sensitivity |
|  | Neighbors of bacterial load |
|  | Neighbors of cancer survival |
|  | Neighbors of graft survival |
|  | Neighbors of therapeutic efficacy |
|  | Neighbors of lethality |
|  | Neighbors of tumor regression |
|  | Neighbors of recurrence-free survival |
|  | Neighbors of tumor response |
|  | Neighbors of long-term survival |
|  | Neighbors of treatment outcome |
|  | Neighbors of bone mass |
|  | Neighbors of body weight |
|  | Neighbors of body weight gain |
|  | Neighbors of cardiovascular risk |
|  | Neighbors of tumor promotion |
|  | Neighbors of neutrophil count |
|  | Neighbors of glucose tolerance |
|  | Neighbors of morbidity |
|  | Neighbors of fibrosis progression |
|  | Neighbors of mortality rate |
|  | Neighbors of fat mass |
|  | Neighbors of exhaustion |
|  | Neighbors of bone mineral density |
|  | Neighbors of adverse outcome |
|  | Neighbors of radioresistance |
|  | Neighbors of cell density |
|  | Neighbors of progression-free survival |
|  | Neighbors of apoptosis rate |
|  | Neighbors of early mortality |
|  | Neighbors of shear stress |
|  | Neighbors of viral load |
|  | Neighbors of blood pressure |
|  | Neighbors of leukocyte count |
|  | Neighbors of vascular reactivity |
|  | Neighbors of vascular leakage |
|  | Neighbors of body mass index |
|  | Neighbors of vascular density |
|  | Neighbors of muscle mass |
|  | Neighbors of cancer risk |
|  | Neighbors of cancer incidence |
|  | Neighbors of systolic blood pressure |
|  | Neighbors of blood glucose |
|  | Neighbors of renal hypertrophy |
|  | Neighbors of fasting plasma glucose |
|  | Neighbors of osteoclast number |
|  | Neighbors of cancer recurrence |
|  | Neighbors of energy expenditure |
|  | Neighbors of glucose level |
|  | Neighbors of vascular tone |
|  | Neighbors of left ventricular ejection fraction |
|  | Neighbors of breast cancer risk |
|  | Neighbors of cell permeability |
|  | Neighbors of pregnancy outcome |
|  | Neighbors of functional status |
|  | Neighbors of gastric cancer risk |
|  | Neighbors of neurological outcome |
|  | Neighbors of infarct area |
|  | Neighbors of fertility |
|  | Neighbors of recurrence rate |
|  | Neighbors of pathological response |
|  | Neighbors of monocyte count |
|  | Neighbors of metabolic response |
|  | Neighbors of insulin level |
|  | Neighbors of comorbidity |
|  | Neighbors of bone volume |
|  | Neighbors of fibroblast phenotype |
|  | Neighbors of fractional shortening |
|  | Neighbors of disease exacerbation |
|  | Neighbors of tumor progression |
|  | Neighbors of cancer progression |
|  | Neighbors of inflammatory status |
|  | Neighbors of overall survival |
|  | Neighbors of disease severity |
|  | Neighbors of tumor size |
|  | Neighbors of lesion size |
|  | Neighbors of mortality |
|  | Neighbors of survival rate |
|  | Neighbors of infarct size |
|  | Neighbors of survival time |
|  | Neighbors of functional recovery |
|  | Neighbors of insulin sensitivity |
|  | Neighbors of bacterial load |
|  | Neighbors of cancer survival |
|  | Neighbors of graft survival |
|  | Neighbors of therapeutic efficacy |
|  | Neighbors of lethality |
|  | Neighbors of tumor regression |
|  | Neighbors of recurrence-free survival |
|  | Neighbors of tumor response |
|  | Neighbors of long-term survival |
|  | Neighbors of treatment outcome |
|  | Neighbors of bone mass |
|  | Neighbors of body weight |
|  | Neighbors of body weight gain |
|  | Neighbors of cardiovascular risk |
|  | Neighbors of tumor promotion |
|  | Neighbors of neutrophil count |
|  | Neighbors of glucose tolerance |
|  | Neighbors of morbidity |
|  | Neighbors of fibrosis progression |
|  | Neighbors of mortality rate |
|  | Neighbors of fat mass |
|  | Neighbors of exhaustion |
|  | Neighbors of bone mineral density |
|  | Neighbors of adverse outcome |
|  | Neighbors of radioresistance |
|  | Neighbors of cell density |
|  | Neighbors of progression-free survival |
|  | Neighbors of apoptosis rate |
|  | Neighbors of early mortality |
|  | Neighbors of shear stress |
|  | Neighbors of viral load |
|  | Neighbors of blood pressure |
|  | Neighbors of leukocyte count |
|  | Neighbors of vascular reactivity |
|  | Neighbors of vascular leakage |
|  | Neighbors of body mass index |
|  | Neighbors of vascular density |
|  | Neighbors of muscle mass |
|  | Neighbors of cancer risk |
|  | Neighbors of cancer incidence |
|  | Neighbors of systolic blood pressure |
|  | Neighbors of blood glucose |
|  | Neighbors of renal hypertrophy |
|  | Neighbors of fasting plasma glucose |
|  | Neighbors of osteoclast number |
|  | Neighbors of cancer recurrence |
|  | Neighbors of energy expenditure |
|  | Neighbors of glucose level |
|  | Neighbors of vascular tone |
|  | Neighbors of left ventricular ejection fraction |
|  | Neighbors of breast cancer risk |
|  | Neighbors of cell permeability |
|  | Neighbors of pregnancy outcome |
|  | Neighbors of functional status |
|  | Neighbors of gastric cancer risk |
|  | Neighbors of neurological outcome |
|  | Neighbors of infarct area |
|  | Neighbors of fertility |
|  | Neighbors of recurrence rate |
|  | Neighbors of pathological response |
|  | Neighbors of monocyte count |
|  | Neighbors of metabolic response |
|  | Neighbors of insulin level |
|  | Neighbors of comorbidity |
|  | Neighbors of bone volume |
|  | Neighbors of fibroblast phenotype |
|  | Neighbors of fractional shortening |
|  | Neighbors of cell development |
|  | Neighbors of immune response |
|  | Neighbors of cell population |
|  | Neighbors of T-cell development |
|  | Neighbors of inflammatory response |
|  | Neighbors of wound healing |
|  | Neighbors of angiogenesis |
|  | Neighbors of innate immune response |
|  | Neighbors of cell function |
|  | Neighbors of macrophage activation |
|  | Neighbors of cell formation |
|  | Neighbors of adaptive immune response |
|  | Neighbors of osteoclast development |
|  | Neighbors of epithelial to mesenchymal transition |
|  | Neighbors of immunity |
|  | Neighbors of cell proliferative response |
|  | Neighbors of chemotaxis |
|  | Neighbors of dendritic cell differentiation |
|  | Neighbors of cell survival |
|  | Neighbors of osteoclast differentiation |
|  | Neighbors of cell phenotype |
|  | Neighbors of T-cell proliferation |
|  | Neighbors of tissue remodeling |
|  | Neighbors of cell adhesion |
|  | Neighbors of T-cell activation |
|  | Neighbors of endothelial cell proliferation |
|  | Neighbors of fibrogenesis |
|  | Neighbors of tumor growth |
|  | Neighbors of dendritic cell development |
|  | Neighbors of tissue repair |
|  | Neighbors of cell count |
|  | Neighbors of cell infiltration |
|  | Neighbors of cell interaction |
|  | Neighbors of SMC proliferation |
|  | Neighbors of lumen formation |
|  | Neighbors of endothelial cell migration |
|  | Neighbors of adipogenesis |
|  | Neighbors of lipid storage |
|  | Neighbors of cell damage |
|  | Neighbors of pregnancy |
|  | Neighbors of monocyte differentiation |
|  | Neighbors of aging |
|  | Neighbors of cell motility |
|  | Neighbors of bone resorption |
|  | Neighbors of monocyte migration |
|  | Neighbors of fibroblast proliferation |
|  | Neighbors of adipocyte differentiation |
|  | Neighbors of macrophage migration |
|  | Neighbors of microglial activation |
|  | Neighbors of neutrophil recruitment |
|  | Neighbors of regeneration |
|  | Neighbors of cell migration |
|  | Neighbors of cancer growth |
|  | Neighbors of stem cell proliferation |
|  | Neighbors of smooth muscle cell migration |
|  | Neighbors of hepatic regeneration |
|  | Neighbors of transendothelial migration |
|  | Neighbors of phagocytosis |
|  | Neighbors of immune system activation |
|  | Neighbors of leukocyte migration |
|  | Neighbors of leukocyte recruitment |
|  | Neighbors of osteoclast formation |
|  | Neighbors of monocyte recruitment |
|  | Neighbors of cell invasion |
|  | Neighbors of heart function |
|  | Neighbors of hemopoiesis |
|  | Neighbors of bone remodeling |
|  | Neighbors of ossification |
|  | Neighbors of T-cell function |
|  | Neighbors of immune system function |
|  | Neighbors of lung development |
|  | Neighbors of autophagy |
|  | Neighbors of monocyte adhesion |
|  | Neighbors of transcription activation |
|  | Neighbors of senescence |
|  | Neighbors of T-cell response |
|  | Neighbors of tumor immunity |
|  | Neighbors of hepatocyte proliferation |
|  | Neighbors of cell transdifferentiation |
|  | Neighbors of neuroprotection |
|  | Neighbors of stem cell differentiation |
|  | Neighbors of endothelial cell function |
|  | Neighbors of neuronal death |
|  | Neighbors of cell growth |
|  | Neighbors of ROS generation |
|  | Neighbors of epithelial cell proliferation |
|  | Neighbors of first trimester pregnancy |
|  | Neighbors of cancer cell growth |
|  | Neighbors of sensitization |
|  | Neighbors of hemato-encephalic barrier |
|  | Neighbors of macrophage function |
|  | Neighbors of cellular immune response |
|  | Neighbors of cellular senescence |
|  | Neighbors of colony formation |
|  | Neighbors of macrophage differentiation |
|  | Neighbors of cell differentiation |
|  | Neighbors of keratinocyte proliferation |
|  | Neighbors of stem cell migration |
|  | Neighbors of life span |
|  | Neighbors of cell homeostasis |
|  | Neighbors of psoriasis |
|  | Neighbors of inflammatory disease |
|  | Neighbors of rheumatoid arthritis |
|  | Neighbors of atherosclerosis |
|  | Neighbors of fibrosis |
|  | Neighbors of colitis |
|  | Neighbors of arthritis |
|  | Neighbors of inflammatory bowel disease |
|  | Neighbors of injury |
|  | Neighbors of atherogenesis |
|  | Neighbors of vascular remodeling |
|  | Neighbors of metastasis |
|  | Neighbors of carcinogenesis |
|  | Neighbors of multiple sclerosis |
|  | Neighbors of chronic inflammation |
|  | Neighbors of inflammation |
|  | Neighbors of tumor microenvironment |
|  | Neighbors of pathological angiogenesis |
|  | Neighbors of obesity |
|  | Neighbors of vascular disease |
|  | Neighbors of diabetes mellitus |
|  | Neighbors of asthma |
|  | Neighbors of pneumonia |
|  | Neighbors of hyperplasia |
|  | Neighbors of osteoarthritis |
|  | Neighbors of liver fibrosis |
|  | Neighbors of chronic obstructive pulmonary disease |
|  | Neighbors of myocardial infarction |
|  | Neighbors of leukocyte infiltration |
|  | Neighbors of reperfusion injury |
|  | Neighbors of airway inflammation |
|  | Neighbors of hepatitis |
|  | Neighbors of insulin resistance |
|  | Neighbors of infection |
|  | Neighbors of vascular injury |
|  | Neighbors of renal injury |
|  | Neighbors of preeclampsia |
|  | Neighbors of macrophage infiltration |
|  | Neighbors of liver injury |
|  | Neighbors of neuroinflammation |
|  | Neighbors of sepsis |
|  | Neighbors of death |
|  | Neighbors of type 1 diabetes |
|  | Neighbors of encephalomyelitis |
|  | Neighbors of autoimmune disease |
|  | Neighbors of ischemic stroke |
|  | Neighbors of dermatitis |
|  | Neighbors of neutrophil infiltration |
|  | Neighbors of heart disease |
|  | Neighbors of diabetic nephropathy |
|  | Neighbors of lung injury |
|  | Neighbors of immunopathology |
|  | Neighbors of systemic sclerosis |
|  | Neighbors of hepatocellular carcinoma |
|  | Neighbors of autoimmunity |
|  | Neighbors of acute lung injury |
|  | Neighbors of gastric cancer |
|  | Neighbors of atherosclerotic plaque |
|  | Neighbors of experimental autoimmune encephalomyelitis |
|  | Neighbors of colorectal cancer |
|  | Neighbors of cardiac remodeling |
|  | Neighbors of breast cancer |
|  | Neighbors of ischemia |
|  | Neighbors of systemic lupus erythematosus |
|  | Neighbors of melanoma |
|  | Neighbors of collagen-induced arthritis |
|  | Neighbors of cartilage degeneration |
|  | Neighbors of interstitial fibrosis |
|  | Neighbors of neoplasm invasion |
|  | Neighbors of kidney disease |
|  | Neighbors of heart failure |
|  | Neighbors of lung disease |
|  | Neighbors of pulmonary fibrosis |
|  | Neighbors of cardiovascular disease |
|  | Neighbors of stroke |
|  | Neighbors of cancer |
|  | Neighbors of cerebral ischemia |
|  | Neighbors of type 2 diabetes |
|  | Neighbors of neoplasm |
|  | Neighbors of endometriosis |
|  | Neighbors of neointima |
|  | Neighbors of vasculitis |
|  | Neighbors of atrophy |
|  | Neighbors of diet-induced obesity |
|  | Neighbors of respiratory hypersensitivity |
|  | Neighbors of myocardial fibrosis |
|  | Neighbors of synovitis |
|  | Neighbors of coronary artery disease |
|  | Neighbors of rupture |
|  | Neighbors of endothelial cell dysfunction |
|  | Neighbors of liver disease |
|  | Neighbors of prostate cancer |
|  | Neighbors of hypertrophy |
|  | Neighbors of osteolysis |
|  | Neighbors of ovarian cancer |
|  | Neighbors of pancreatic cancer |
|  | Neighbors of hypertension |
|  | Neighbors of intimal hyperplasia |
|  | Neighbors of allograft rejection |
|  | Neighbors of proteinuria |
|  | Neighbors of epithelial cell |
|  | Neighbors of keratinocyte |
|  | Neighbors of vascular smooth muscle cell |
|  | Neighbors of umbilical vein endothelial cell |
|  | Neighbors of monocyte |
|  | Neighbors of endometrial stromal cell |
|  | Neighbors of alveolar macrophage |
|  | Neighbors of synoviocyte |
|  | Neighbors of macrophage |
|  | Neighbors of adipocyte |
|  | Neighbors of activated monocyte |
|  | Neighbors of fibroblast |
|  | Neighbors of airway epithelial cell |
|  | Neighbors of PBMC |
|  | Neighbors of stromal cell |
|  | Neighbors of airway smooth muscle cell |
|  | Neighbors of osteoblast |
|  | Neighbors of pneumocyte |
|  | Neighbors of endometrium cell |
|  | Neighbors of microglia |
|  | Neighbors of trophoblast |
|  | Neighbors of intestine epithelium cell |
|  | Neighbors of mononuclear cell |
|  | Neighbors of hepatic stellate cell |
|  | Neighbors of hepatocyte |
|  | Neighbors of decidual cell |
|  | Neighbors of kidney mesangial cell |
|  | Neighbors of endothelial cell |
|  | Neighbors of dendritic cell |
|  | Neighbors of Kuppfer cell |
|  | Neighbors of proadipocyte |
|  | Neighbors of kidney tubule cell |
|  | Neighbors of inflammatory cell |
|  | Neighbors of bone marrow macrophage |
|  | Neighbors of peritoneal macrophage |
|  | Neighbors of osteoclast |
|  | Neighbors of neutrophil |
|  | Neighbors of smooth muscle myocyte |
|  | Neighbors of astrocyte |
|  | Neighbors of bone marrow derived dendritic cell |
|  | Neighbors of lung fibroblast |
|  | Neighbors of activated T-cell |
|  | Neighbors of mesothelial cell |
|  | Neighbors of mononuclear phagocyte |
|  | Neighbors of capillary endothelial cell |
|  | Neighbors of immunocompetent cell |
|  | Neighbors of granulocyte |
|  | Neighbors of cardiac myocyte |
|  | Neighbors of cancer-associated fibroblast |
|  | Neighbors of myeloid cell |
|  | Neighbors of mesenchymal stroma cell |
|  | Neighbors of corneal epithelial cell |
|  | Neighbors of cytotrophoblast |
|  | Neighbors of stem cell |
|  | Neighbors of tumor infiltrating macrophage |
|  | Neighbors of sinusoidal cell |
|  | Neighbors of B-cell |
|  | Neighbors of granulosa cell |
|  | Neighbors of leukocyte |
|  | Neighbors of chondrocyte |
|  | Neighbors of insulin-secreting cell |
|  | Neighbors of mesenchymal cell |
|  | Neighbors of T-cell |
|  | Neighbors of M1 macrophage |
|  | Neighbors of myofibroblast |
|  | Neighbors of pancreatic stellate cell |
|  | Neighbors of mast cell |
|  | Neighbors of bone marrow stromal cell |
|  | Neighbors of spleen cell |
|  | Neighbors of brain capillary endothelial cell |
|  | Neighbors of neuroglia |
|  | Neighbors of thyroid epithelial cell |
|  | Neighbors of aortic smooth muscle cell |
|  | Neighbors of hematopoietic cell |
|  | Neighbors of adipose macrophage |
|  | Neighbors of podocyte |
|  | Neighbors of mammary epithelial cell |
|  | Neighbors of conventional dendritic cell |
|  | Neighbors of pericyte |
|  | Neighbors of antigen-presenting cell |
|  | Neighbors of epidermis cell |
|  | Neighbors of bone marrow derived mast cell |
|  | Neighbors of phagocyte |
|  | Neighbors of kidney cell |
|  | Neighbors of syncytiotrophoblast |
|  | Neighbors of lymphocyte |
|  | Neighbors of Langerhans cell |
|  | Neighbors of decidual natural killer cell |
|  | Neighbors of thymic epithelial cell |
|  | Neighbors of brain cell |
|  | Neighbors of M2 macrophage |
|  | Neighbors of peritoneum cell |
|  | Neighbors of lymphoid cell |
|  | Neighbors of retina cell |
|  | Neighbors of blood cell |
|  | Neighbors of skin cell |
|  | Neighbors of luteal cell |
|  | Neighbors of peripheral lymphocyte |
|  | Neighbors of activated B-cell |
|  | Neighbors of lymph node cell |
|  | Neighbors of lung |
|  | Neighbors of liver |
|  | Neighbors of kidney |
|  | Neighbors of skin |
|  | Neighbors of intestine |
|  | Neighbors of lymph node |
|  | Neighbors of colon |
|  | Neighbors of airway |
|  | Neighbors of heart |
|  | Neighbors of aorta |
|  | Neighbors of gastrointestinal tract |
|  | Neighbors of microvessel |
|  | Neighbors of bone |
|  | Neighbors of placenta |
|  | Neighbors of synovial membrane |
|  | Neighbors of uterus |
|  | Neighbors of esophagus |
|  | Neighbors of periodontium |
|  | Neighbors of retina |
|  | Neighbors of brain |
|  | Neighbors of ovary |
|  | Neighbors of breast |
|  | Neighbors of blood vessel wall |
|  | Neighbors of stomach |
|  | Neighbors of islets of Langerhans |
|  | Neighbors of joint |
|  | Neighbors of cardiovascular system |
|  | Neighbors of pancreas |
|  | Neighbors of prostate |
|  | Neighbors of central nervous system |
|  | Neighbors of kidney glomerulus |
|  | Neighbors of lymphatic system |
|  | Neighbors of colorectal region |
|  | Neighbors of decidua |
|  | Neighbors of amnion |
|  | Neighbors of immune system |
|  | Neighbors of spleen |
|  | Neighbors of peritoneum |
|  | Neighbors of allantois |
|  | Neighbors of coronary artery |
|  | Neighbors of left ventricle |
|  | Neighbors of uterine cervix |
|  | Neighbors of vein |
|  | Neighbors of arteriole |
|  | Neighbors of villus |
|  | Neighbors of nose |
|  | Neighbors of eye |
|  | Neighbors of cornea |
|  | Neighbors of hippocampus |
|  | Neighbors of intervertebral disc |
|  | Neighbors of lymph node of mesentery |
|  | Neighbors of mammary gland |
|  | Neighbors of vagina |
|  | Neighbors of ankle joint |
|  | Neighbors of vitreous body |
|  | Neighbors of thymus gland |
|  | Neighbors of cerebral cortex |
|  | Neighbors of brain blood vessel |
|  | Neighbors of myelin sheath |
|  | Neighbors of bladder |
|  | Neighbors of nervous system |
|  | Neighbors of blastocyst |
|  | Neighbors of nasopharynx |
|  | Neighbors of periodontal ligament |
|  | Neighbors of corpus luteum of ovary |
|  | Neighbors of sympathetic nervous system |
|  | Neighbors of nerve |
|  | Neighbors of endocrine system |
|  | Neighbors of globus pallidus |
|  | Neighbors of lymphatic vessels |
|  | Neighbors of pleura |
|  | Neighbors of Fallopian tube |
|  | Neighbors of hypothalamus |
|  | Neighbors of choroid plexus |
|  | Neighbors of NF-kB family |
|  | Neighbors of oxidized LDL |
|  | Neighbors of JNK |
|  | Neighbors of IL1 family |
|  | Neighbors of PPAR |
|  | Neighbors of Jun/Fos |
|  | Neighbors of NFKBI |
|  | Neighbors of PKC |
|  | Neighbors of PI3K |
|  | Neighbors of STAT family |
|  | Neighbors of mitogen-activated protein kinase |
|  | Neighbors of IFNAR ligand |
|  | Neighbors of transforming growth factor |
|  | Neighbors of TLR |
|  | Neighbors of cytokine |
|  | Neighbors of interferon |
|  | Neighbors of interleukin |
|  | Neighbors of JAK |
|  | Neighbors of MEK1/2 |
|  | Neighbors of PDGF |
|  | Neighbors of Ras GTPase |
|  | Neighbors of ERK1/2 |
|  | Neighbors of growth factor |
|  | Neighbors of PKA |
|  | Neighbors of ovalbumin |
|  | Neighbors of LDL |
|  | Neighbors of NF-AT family |
|  | Neighbors of beta adrenoceptor |
|  | Neighbors of chorionic gonadotropin |
|  | Neighbors of IKBK |
|  | Neighbors of adipokine |
|  | Neighbors of protein tyrosine kinase |
|  | Neighbors of NAD(P)H oxidase |
|  | Neighbors of IgG |
|  | Neighbors of SMAD subfamily |
|  | Neighbors of AMPK |
|  | Neighbors of LXR |
|  | Neighbors of HDL |
|  | Neighbors of p38 |
|  | Neighbors of inflammatory cytokine |
|  | Neighbors of IL23 |
|  | Neighbors of estrogen receptor |
|  | Neighbors of T-cell receptor |
|  | Neighbors of histone deacetylase |
|  | Neighbors of PDGF-BB |
|  | Neighbors of IL1R |
|  | Neighbors of HIF-1 |
|  | Neighbors of GPCR |
|  | Neighbors of IL12 |
|  | Neighbors of Notch |
|  | Neighbors of heat shock protein 90 |
|  | Neighbors of lectin |
|  | Neighbors of fibrinogen |
|  | Neighbors of aldehyde reductase |
|  | Neighbors of interleukin-35 |
|  | Neighbors of TSH |
|  | Neighbors of Ang II receptor |
|  | Neighbors of superoxide dismutase |
|  | Neighbors of proteasome endopeptidase complex |
|  | Neighbors of class nuclear receptor with C4 zinc fingers |
|  | Neighbors of histone H3 |
|  | Neighbors of alpha2 adrenoceptor |
|  | Neighbors of vitronectin receptor (integrin) |
|  | Neighbors of PP2A |
|  | Neighbors of retinoid-X receptor subfamily |
|  | Neighbors of SAPK |
|  | Neighbors of WNT |
|  | Neighbors of gonadotropins |
|  | Neighbors of immunoglobulin |
|  | Neighbors of PP2B |
|  | Neighbors of phospholipase |
|  | Neighbors of ITG |
|  | Neighbors of endostatins |
|  | Neighbors of CD3 complex |
|  | Neighbors of relaxin |
|  | Neighbors of TLR1/2 heterodimer |
|  | Neighbors of TORC1 |
|  | Neighbors of RA receptor |
|  | Neighbors of histone |
|  | Neighbors of Rho kinase |
|  | Neighbors of neuropeptides |
|  | Neighbors of pancreatic elastase |
|  | Neighbors of adrenoceptor |
|  | Neighbors of phosphodiesterase |
|  | Neighbors of caspase |
|  | Neighbors of deacetylase |
|  | Neighbors of TLR4/Myd88 |
|  | Neighbors of TNF-alpha receptor |
|  | Model of PPARG signaling in psoriasis |
|  | PPARG negative regulators and targets |
|  | Model of PPARG related pathways in psoriasis (short version) |
|  | sars-cov no binding |
|  | dsRNA induces innate immunity and INFs in alveolar cells |
|  | Dendritic Cell Activation in COVID19 |
|  | Figure 2\_Levothyroxine and aspirin effects crossing |
|  | Figure 2\_Levothyroxine and aspirin effects crossing |
|  | Overview. Diseases of the digestive system (chapter 10) |
|  | 1\_Inflammation in keratinocytes in the pathology of acne |
|  | 1\_Differentiation of psoriatic T cells |
|  | 1\_TNF and IL1B provoke ECM degradation in osteoarthritis |
|  | 2\_Chondrocyte death in osteoarthritis |
|  | 1\_The role of Toll-like receptors in pyelonephritis |
|  | 2\_The role of endothelial cells in pyelonephritis |
|  | 1\_Local estrogen production stimulates ectopic endometrium proliferation |
|  | 1\_Insufficient activation of immune response in the middle ear epithelium cells in otitis media |
|  | 2\_Pathogens stimulate mucins expression in the middle ear |
|  | 1\_Cigarette smoke causes dysfunction and production of inflammatory mediators in alveolar epithelial cells |
|  | 3\_Role of alveolar macrophages in pulmonary tissue destruction in COPD |
|  | 1\_Th2 cell response in asthma |
|  | 3\_1\_Mucus accumulation in asthma |
|  | 4\_Mucin hyperproduction in goblet and mucous cells in COPD |
|  | 2\_Paneth cell dysfunction in Crohn's disease |
|  | 1\_Defects in response to pathogens in the gut promotes inflammation in Crohn's disease |
|  | 3\_Leukocyte migration toward the endothelial cells in the intestine microvasculature |
|  | 2\_Intestine epithelial cells drive immunological responses in ulcerative colitis |
|  | 4\_1\_Polymorphisms associated with inflammatory bowel diseases |
|  | 2\_Lipotoxicity in NAFLD |
|  | 5\_Human papilloma virus E6 and E7 proteins promote epithelial cell proliferation |
|  | 3\_Impairment of iron release from splenic macrophages |
|  | 5\_The rationale for the treatment of anemia of chronic disease with erythropoietin |
|  | 1\_CFTR expression and degradation in epithelial cells in cystic fibrosis (class I-II mutations) |
|  | 1\_Antigen-presenting cells promote islet dysfunction and immune system activation in T1D |
|  | 3\_Islet beta-cell destruction in T1D |
|  | Figure 4\_Diabetes mellitus Type 1: conventional dendritic cell |
|  | drug-target pairs direct regulation |
|  | drug-target moltransport |
|  | drug-target molsynthesis |
|  | Differentiation of psoriatic T cells |

---

|  |  |
| --- | --- |
| Group | Genes Associated with Systemic Lupus Erythematosus |
|  | Genes with Mutations Associated with Graves Ophthalmopathy |
|  | Genes with Polymorphisms Associated with Migraine |
|  | Genes with Mutations Associated with Multiple Sclerosis |
|  | other partners for SARS |

---

|  |  |
| --- | --- |
| MedScan ID | 3553 |

---

|  |  |
| --- | --- |
| LocusLink ID | 3553 |
|  | 16176 |
|  | 24494 |

---

|  |  |
| --- | --- |
| Alias | pro-interleukin-1-beta |
|  | OTTHUMP00000203695 |
|  | OTTHUMP00000203694 |
|  | IL1B |
|  | interleukin 1B |
|  | OTTHUMP00000203693 |
|  | preinterleukin 1 beta |
|  | Interleukin IL1 beta |
|  | IL1B\_HUMAN |
|  | RP23-384K11.2 |
|  | beta interferon inducing 22 k factor |
|  | Catabolin |
|  | IL1F2 |
|  | interleukin IL1B |
|  | OTTMUSP00000016525 |
|  | interleukin IL1F2 |
|  | IL1beta |
|  | interleukin 1 beta |
|  | interferon beta inducing 22k factor |
|  | interleukin IL1B\_HUMAN |
|  | IL-1 |
|  | IL1-BETA |
|  | IL-1 beta |
|  | interleukin-1 beta |
|  | Il-1b |
|  | IL-1beta |
|  | IL-1F2 |
|  | interleukin 1, beta |

---

|  |  |
| --- | --- |
| GO ID | 0005125 |
|  | 0005178 |
|  | 0005149 |
|  | 0019904 |
|  | 0000165 |
|  | 0000187 |
|  | 0006915 |
|  | 0048143 |
|  | 0007267 |
|  | 0035690 |
|  | 0071222 |
|  | 0071260 |
|  | 0071407 |
|  | 0071310 |
|  | 0019221 |
|  | 0035234 |
|  | 0007566 |
|  | 0097192 |
|  | 0001660 |
|  | 0030213 |
|  | 0006955 |
|  | 0006954 |
|  | 0032611 |
|  | 0070498 |
|  | 0032635 |
|  | 0031663 |
|  | 0070487 |
|  | 0043407 |
|  | 0070164 |
|  | 0008285 |
|  | 2001240 |
|  | 0010829 |
|  | 0046627 |
|  | 0050995 |
|  | 0045833 |
|  | 0050768 |
|  | 0050805 |
|  | 0030593 |
|  | 0051091 |
|  | 0043123 |
|  | 0046330 |
|  | 0051092 |
|  | 1901224 |
|  | 1902680 |
|  | 0002711 |
|  | 0042102 |
|  | 2000556 |
|  | 0045766 |
|  | 0060559 |
|  | 0060355 |
|  | 0051781 |
|  | 0030335 |
|  | 0008284 |
|  | 0045080 |
|  | 0045917 |
|  | 0010718 |
|  | 0031622 |
|  | 0010628 |
|  | 0060252 |
|  | 0032725 |
|  | 0034116 |
|  | 0035066 |
|  | 0033129 |
|  | 0050729 |
|  | 0032729 |
|  | 0045086 |
|  | 0045410 |
|  | 0032755 |
|  | 2000778 |
|  | 0032757 |
|  | 0050996 |
|  | 0051044 |
|  | 0045840 |
|  | 0071639 |
|  | 0035505 |
|  | 0150078 |
|  | 0045429 |
|  | 1900745 |
|  | 0050766 |
|  | 0031394 |
|  | 0032308 |
|  | 0046827 |
|  | 0001934 |
|  | 0045944 |
|  | 0045893 |
|  | 0010575 |
|  | 0030949 |
|  | 0043491 |
|  | 0070372 |
|  | 0043122 |
|  | 0050691 |
|  | 1903140 |
|  | 0050796 |
|  | 0050767 |
|  | 0050999 |
|  | 0033198 |
|  | 0009743 |
|  | 0070555 |
|  | 0032496 |
|  | 0030730 |
|  | 0007165 |
|  | 0014805 |
|  | 0005829 |
|  | 0070062 |
|  | 0005576 |
|  | 0005615 |
|  | 0005764 |
|  | 0030141 |
|  | 0050900 |
|  | 0007613 |
|  | 2000173 |
|  | 0010629 |
|  | 0014050 |
|  | 2000178 |
|  | 0045665 |
|  | 0000122 |
|  | 0070374 |
|  | 0043507 |
|  | 0043065 |
|  | 0048711 |
|  | 0010942 |
|  | 0007204 |
|  | 0045687 |
|  | 0033092 |
|  | 0043525 |
|  | 0090023 |
|  | 0032874 |
|  | 0035176 |
|  | 0005737 |
|  | 0031410 |
|  | 0031982 |
|  | 0002526 |
|  | 0071236 |
|  | 0071398 |
|  | 0071333 |
|  | 0071396 |
|  | 0002439 |
|  | 0007611 |
|  | 0030728 |
|  | 0010469 |
|  | 0033591 |
|  | 0071548 |
|  | 0042493 |
|  | 0032355 |
|  | 0045471 |
|  | 0010332 |
|  | 0051384 |
|  | 0009408 |
|  | 0001666 |
|  | 0035902 |
|  | 0043278 |
|  | 0007584 |
|  | 0014070 |
|  | 0010243 |
|  | 0010193 |
|  | 0043434 |
|  | 0035634 |
|  | 0033280 |
|  | 0042060 |
|  | 0005776 |
|  | 0002437 |
|  | 2000866 |
|  | 0005622 |
|  | 0005623 |
|  | 0071414 |
|  | 0043066 |
|  | 0008210 |
|  | 0005102 |
|  | 0019742 |
|  | 0001525 |
|  | 0030638 |
|  | 0006144 |
|  | 0007568 |
|  | 0010977 |
|  | 0006916 |
|  | 0009100 |
|  | 0036273 |
|  | 0036270 |
|  | 0042221 |
|  | 0042346 |
|  | 0010552 |
|  | 0008083 |

---

|  |  |
| --- | --- |
| KEGG ID | hsa:3553 |
|  | mmu:16176 |
|  | rno:24494 |

---

|  |  |
| --- | --- |
| Organism | Homo sapiens {Organism urn:agi-taxid:9606} |
|  | Mus musculus {Organism urn:agi-taxid:10090} |
|  | Rattus norvegicus {Organism urn:agi-taxid:10116} |
|  | Homo sapiens |
|  | Mus musculus |
|  | Rattus norvegicus |

---

|  |  |
| --- | --- |
| Mouse chromosome position | 2 62.97 cM |
|  | 2 73.0 cM |

---

|  |  |
| --- | --- |
| OMIM ID | 147720 |
|  | 137215 |

---

|  |  |
| --- | --- |
| Rat chromosome position | 3q36 |

---

|  |  |
| --- | --- |
| Hugo ID | 5992 |
|  | HGNC:5992 |

---

|  |  |
| --- | --- |
| Human chromosome position | 2q14.1 |
|  | 2q14 |

---

|  |  |
| --- | --- |
| Swiss-Prot Accession | P01584 |
|  | P01584.2 |
|  | P10749 |
|  | P10749.1 |
|  | Q5BKB0 |
|  | Q63264.1 |
|  | Q53X59 |
|  | Q53XX2 |
|  | Q7M4S7 |
|  | Q7RU01 |
|  | Q96HE5 |
|  | Q9UCT6 |
|  | Q2M4J6 |
|  | O43645 |
|  | Q63264 |
|  | Q3TI17 |

---

|  |  |
| --- | --- |
| PIR ID | A21851 |
|  | A25542 |
|  | I55969 |

---

|  |  |
| --- | --- |
| GenBank ID | NC\_000002 |
|  | NM\_000576 |
|  | NP\_000567 |
|  | XM\_017003988 |
|  | XP\_016859477 |
|  | NG\_008851 |
|  | AC079753 |
|  | AAX88888 |
|  | AY137079 |
|  | AAM88883 |
|  | BN000002 |
|  | CAD29872 |
|  | CH471217 |
|  | EAW73605 |
|  | EAW73606 |
|  | EAW73607 |
|  | JA104597 |
|  | CCA63968 |
|  | LC333978 |
|  | M15840 |
|  | AAA74137 |
|  | X04500 |
|  | CAA28185 |
|  | X52430 |
|  | X52431 |
|  | AB451494 |
|  | BAG70308 |
|  | AF043335 |
|  | AAC03536 |
|  | AK313983 |
|  | BC008678 |
|  | AAH08678 |
|  | BT007213 |
|  | AAP35877 |
|  | CR407679 |
|  | CAG28607 |
|  | K02770 |
|  | AAA36106 |
|  | M15330 |
|  | AAA59135 |
|  | M54933 |
|  | AAA59136 |
|  | X02532 |
|  | CAA26372 |
|  | X56087 |
|  | CAA39567 |
|  | P01584 |
|  | NC\_000068 |
|  | XM\_006498795 |
|  | XP\_006498858 |
|  | NM\_008361 |
|  | NP\_032387 |
|  | AL808143 |
|  | AY902319 |
|  | AAX90604 |
|  | CH466519 |
|  | EDL28238 |
|  | U03987 |
|  | X04964 |
|  | CAA28637 |
|  | AK156396 |
|  | AK157245 |
|  | AK168047 |
|  | BAE40029 |
|  | AK224980 |
|  | AK225002 |
|  | AK225003 |
|  | BC011437 |
|  | AAH11437 |
|  | M15131 |
|  | AAA39276 |
|  | P10749 |
|  | NC\_005102 |
|  | NM\_031512 |
|  | NP\_113700 |
|  | AC\_000071 |
|  | AABR07053713 |
|  | AAHX01025010 |
|  | CH473949 |
|  | EDL80163 |
|  | BC091141 |
|  | AAH91141 |
|  | M98820 |
|  | AAA41426 |
|  | Q63264 |
|  | NT\_039207 |
|  | AAHY01020661 |
|  | XP\_006712559 |
|  | ABM84780 |
|  | NW\_047658 |
|  | AC\_000045 |
|  | DQ893854 |
|  | NW\_001084813 |
|  | Q2M4J6 |
|  | Q3TI17 |
|  | Q5BKB0 |
|  | NT\_022135 |
|  | AMYH02004594 |
|  | NW\_001030694 |
|  | AC\_000024 |
|  | AABR06026121 |
|  | AABR06026120 |
|  | ABM81599 |
|  | O43645 |
|  | CAM22719 |
|  | AC\_000134 |
|  | DQ890673 |
|  | NW\_001838841 |
|  | NC\_018913 |
|  | ABBA01074249 |
|  | NW\_921474 |
|  | XM\_006712496 |

---

|  |  |
| --- | --- |
| Swiss-Prot ID | IL1B\_HUMAN |
|  | IL1B\_MOUSE |
|  | Q5BKB0\_RAT |
|  | Q2M4J6\_MOUSE |

---

|  |  |
| --- | --- |
| Cell Localization | Cytoplasm |
|  | Cytosol |
|  | Lysosome |
|  | Secreted |
|  | Exosome |
|  | Cytoplasmic vesicle |
|  | Autophagosome |
|  | Cytoplasm, cytosol |
|  | Secreted, exosome |
|  | Cytoplasmic vesicle, autophagosome |
|  | Extracellular |

---

|  |  |
| --- | --- |
| Ensembl ID | ENSG00000125538 |
|  | ENSP00000263341.2 |
|  | ENST00000263341.6 |
|  | ENSMUSG00000027398 |
|  | ENSMUSP00000028881.7 |
|  | ENSMUST00000028881.13 |
|  | ENSRNOG00000004649 |
|  | ENSRNOP00000006308.2 |
|  | ENSRNOT00000006308.4 |
|  | ENSMUST00000028881 |
|  | ENSRNOT00000006308 |
|  | ENSP00000263341 |
|  | ENST00000263341 |
|  | ENSRNOP00000006308 |
|  | ENSMUSP00000028881 |

---

|  |  |
| --- | --- |
| MGI ID | MGI:96543 |
|  | 96543 |

---

|  |  |
| --- | --- |
| RGD ID | 2891 |

---

|  |  |
| --- | --- |
| TCDB ID | 1.A.109.1.2 |

---

|  |  |
| --- | --- |
| Unigene ID | Rn.9869 |
|  | Hs.126256 |
|  | Mm.222830 |

---

|  |  |
| --- | --- |
| Homologene ID | 481 |

---

|  |  |
| --- | --- |
| Shape | Rhomb |

---

|  |  |
| --- | --- |
| IPI ID | IPI00114067 |
|  | IPI00876594 |
|  | IPI00307223 |
|  | IPI00206984 |

---
